# Comparing Different Recording Lengths of Dynamic Cerebral Autoregulation: 5 versus 10 Minutes

**DOI:** 10.1155/2018/7803426

**Published:** 2018-01-31

**Authors:** Nai-Fang Chi, Cheng-Yen Wang, Lung Chan, Han-Hwa Hu

**Affiliations:** ^1^Department of Neurology, School of Medicine, College of Medicine, Taipei Medical University, Taipei, Taiwan; ^2^Department of Neurology, Shuang Ho Hospital, Taipei Medical University, Taipei, Taiwan; ^3^Cerebrovascular Research Center, Taipei Medical University, Taipei, Taiwan

## Abstract

We compared the dynamic cerebral autoregulation (dCA) indices between 5- and 10-minute data lengths by analyzing 37 patients with ischemic stroke and 51 controls in this study. Correlation coefficient (*Mx*) and transfer function analysis were applied for dCA analysis. *Mx* and phase shift in all frequency bands were not significantly different between 5- and 10-minute recordings [mean difference: *Mx* = 0.02; phase shift of very low frequency (0.02–0.07 Hz) = 0.3°, low frequency (0.07–0.20 Hz) = 0.6°, and high frequency (0.20–0.50 Hz) = 0.1°]. However, the gains in all frequency bands of a 5-minute recording were slightly but significantly higher than those of a 10-minute recording (mean difference of gain: very low frequency = 0.05 cm/s/mmHg, low frequency = 0.11 cm/s/mmHg, and high frequency = 0.14 cm/s/mmHg). The intraclass correlation coefficients between all dCA indices of 5- and 10-minute recordings were favorable, especially in *Mx* (0.93), phase shift in very low frequency (0.87), and gain in very low frequency (0.94). The areas under the receiver operating characteristic curve for stroke diagnosis between 5- and 10-minute recordings were not different. We concluded that dCA assessed by using a 5-minute recording is not significantly different from that using a 10-minute recording in the clinical application.

## 1. Introduction

Cerebral autoregulation is a physiological mechanism of maintaining a relatively constant cerebral blood flow (CBF) in response to the systemic hemodynamic change. Dynamic cerebral autoregulation (dCA) can be measured by analyzing the correlation between spontaneous or induced changes in CBF and peripheral blood pressure (BP) [1]. The cerebral blood flow velocity (CBFV) recorded by using a transcranial Doppler ultrasonography (TCD) under normocapnia status is a reliable surrogate of CBF [2].

Continuous CBFV and noninvasive BP recordings are commonly used in the studies of dCA. The dCA under spontaneous CBFV and BP changes can be assessed in time domain (correlation coefficient “*Mx*” or autoregulatory index “ARI”) [3, 4] or frequency domain (transfer function analysis, TFA) [5]. Although there is no gold standard method of dCA assessment, it has been proposed that the minimum data recording length is 5 minutes in order to obtain stable results [6]. In past studies, a common recording length of spontaneous CBFV and BP changes is 5 or 10 minutes [5, 7–15] and a longer recording length of more than 20 minutes was also used [16–18]. In subjects with illness, a long recording time will be vulnerable to motion artifacts due to poor cooperation. Therefore, it is better to have a recording time as short as possible in clinical practices. A study revealed that the ARI, *Mx*, and phase shift exhibit large variability under a recording time less than 5 minutes [19]. However, whether a longer recording time more than 5 minutes is beneficial in the research or clinical application is unclear.

This study used both *Mx* and TFA to investigate the agreement between 5 and 10 minutes of recording, as well as comparing their validity for identifying patients with stroke.

## 2. Methods

### 2.1. Subjects

This study was approved by the Institutional Review Board of Taipei Medical University, which comprised the data from our previous study [20] and newly recruited participants. Patients with acute ischemic stroke admitted to the neurology ward of Taipei Medical University Shuang Ho Hospital were consecutively screened for the eligibility of this study. Magnetic resonance imaging and angiography (MRI and MRA), electrocardiography (ECG), extracranial carotid Doppler sonography (ECCD), and transcranial color-coded duplex sonography (TCCS) were the routine exams for each patient with stroke. Patients with atrial fibrillation found in ECG, bilateral poor temporal windows found in TCCS, more than 50% stenosis of internal carotid artery found in ECCD, or more than 50% stenosis of middle cerebral artery (MCA) found in MRA were excluded at initial screening. In the patients who agreed to participate in this study, dCA was measured within 3 months of stroke onset, and stroke severity was measured by using the National Health Institute Stroke Scale (NIHSS) on the day of dCA measurement. Controls without a history of stroke were recruited at the health management center of the same hospital. Written informed consent was obtained from all subjects.

### 2.2. Dynamic Cerebral Autoregulation Measurement and Analysis

The dCA measurements were recorded when the subjects were supine with head elevated at 30° and normal breathing. The end-tidal CO_2_ was measured by using a capnography (Nellcor N85, Medtronic, USA). A TCD monitor (MultiDop-T, DWL, Germany), with 2-MHz probes fixed at temporal region and an insonation depth of 50–60 mm, was used for recording the CBFV in MCA. A finger photoplethysmogram (Finometer Pro, Finapres, the Netherlands), with physiologic calibration (“physiocal”) turned on, was used for recording continuous BP. CBFV and BP of 10 minutes were simultaneously sampled at 50 Hz by using a data acquisition device (NI USB-6221 BNC, National Instruments, USA). Recordings were started after 15 minutes of rest and with a stable end-tidal CO_2_ level. The signals were synchronized between the TCD monitor and Finapres device [20]. The data were inspected manually before dCA analysis, minor artifacts were removed by linear interpolation, and severe artifacts were excluded from analysis.

The raw waveform was downsampled at 10 Hz without detrending, normalization, or filtering for offline analysis. The dCA was analyzed by using *Mx* and TFA. The *Mx* was calculated as the following steps: Pearson correlation coefficients between 20 consecutive 3-second periods (a total of 1 minute) of mean CBFV and BP were calculated, and all correlation coefficients during the recording period were averaged as the *Mx* [4, 21]. *Mx* = 0 indicates intact dCA, which represents that the changes in CBF were independent of those in BP, whereas *Mx* = 1 indicates absent dCA, which represents that the changes in CBF were totally dependent of those in BP [22]. The TFA was performed by using the algorithm provided by the International Cerebral Autoregulation Research Network (CARNet, http://www.car-net.org/content/resources) with its default TFA parameters. The TFA calculated phase shift, gain, and coherence between the CBFV and BP in very low frequency (VLF, 0.02–0.07 Hz), low frequency (LF, 0.07–0.20 Hz), and high frequency (HF, 0.20–0.50 Hz) bands. By using this TFA algorithm, the default data window length is 102 seconds, and a 5-minute recording comprises 5 windows with 50% data overlap, whereas a 10-minute recording comprises 13 windows with 59.9% data overlap. In the transfer function between CBFV and BP, dCA decreases the influence of BP changes on CBFV. In subjects with intact dCA, the changes in CBFV are smaller and are restored faster than those in BP compared to subjects with impaired dCA [6, 22]. Therefore, a large gain and a small phase shift in TFA represent impaired dCA. In patients with cerebrovascular diseases, *Mx* was reported larger than controls [18, 23], and phase shifts were reported smaller than controls [23, 24]. In this study, we compared the dCA calculated from the first 5 minutes, the last 5 minutes, and the total 10 minutes to test the stability and agreement of dCA indices between different recording lengths. We furtherly excluded the patients whose VLF phase shift or gain could not be calculated due to unacceptable low coherence (<0.34 in 5 windows, and <0.14 in 13 windows according to the white paper of CARNet [6]). In patients with a substantially low coherence, the TFA results are unreliable due to poor linear correlation between CBFV and BP, and their *Mx* could be misinterpreted as good dCA [25]. The data of total 88 subjects, including 37 patients with ischemic stroke (age, 56 ± 11 years; 28 males) and 51 controls (age, 47 ± 14 years; 18 males), were enrolled for the final statistical analysis.

### 2.3. Statistical Analysis

The normality of data was checked by using the Shapiro-Wilk test. The normally distributed data were expressed as mean ± standard deviation (SD), and nonnormally distributed data were expressed as median with interquartile range (IQR). The continuous variables between the patients and controls were compared by using the *t*-test or the Mann–Whitney *U* test according to the normality of data. The categorical variables between the patients and controls were compared by using the Fisher's exact test. In the patients with stroke and controls, the dCA of affected side and right side were used for statistical analysis, respectively (the data of controls were from our previous study, in them only the CBFV in right MCA was recorded) [20]. Because most dCA indices were not normally distributed, dCA indices were compared between the first 5-minute, the last 5-minute, and 10-minute recordings by using the Friedman test with post hoc analysis. The agreement and intraindividual correlations between each dCA index from the first 5-minute and 10-minute recordings were tested using the Bland-Altman methods and intraclass correlation coefficient (ICC), respectively. The area under the receiver operating characteristic (ROC) curve of each dCA index was compared between the first 5-minute and 10-minute recordings for identifying patients with stroke in all subjects by using the method proposed by DeLong et al. [26]. *P* < 0.05 was considered statistically significant. Statistical data were analyzed using MedCalc statistical software (version 17.9; MedCalc Software bvba, Ostend, Belgium).

## 3. Results

The clinical characteristics of the subjects are summarized in Table 1. The age, proportion of male sex, hypertension, and diabetes in patients with stroke were significantly higher than those in controls. Most patients had mild stroke severity (NIHSS = 3 ± 3). The agreements between dCA assessed for the first 5 minutes, the last 5 minutes, and 10 minutes are presented in Table 2. All 88 subjects had the results of *Mx* as well as the phase shift and gain in VLF band, but 10 of them did not have the results of phase shift and gain in LF and HF bands due to unacceptable low coherence.

All dCA indices were not significantly different between the first 5 minutes and the last 5 minutes, and the phase shift in all frequency bands and *Mx* were not significantly different between the 5- and 10-minute recordings. However, the gain and coherence in all frequency bands were significantly higher in each of the first and last 5-minute recording than those in the 10-minute recording. The mean difference of each dCA index between the first 5 minutes and 10 minutes calculated by using Bland-Altman methods agreed with the results of Friedman test. This phenomenon existed in both patients and controls (the results of subgroup analysis are presented in the online Supplementary Table (available here)). The ICC of *Mx* and all TFA indices between the first 5- and 10- minute recordings were favorable, especially of *Mx* (0.93), phase shift in VLF (0.87), and gain in VLF (0.94) (Table 2).

The areas under the curve (AUC) of ROC for *Mx*, phase shift, and gain for identifying patients with stroke in all subjects are presented in Table 3. The validity in identifying patients with stroke was favorable for the *Mx* (AUC of the first 5- and 10-minute recordings = 0.714 and 0.719, resp.) and phase shift of VLF (AUC of the first 5- and 10-minute recordings = 0.707 and 0.716, resp.). The AUCs for the phase shift in LF or HF and gain in all frequency bands did not significantly differ from random guesses (AUC = 0.5). The AUCs of all dCA indices between the first 5- and 10-minute recordings were not significantly different. Thus, the validity of dCA indices for identifying patients with stroke was not different between the 5- and 10-minute recordings.

## 4. Discussion

In the current study, all dCA indices remained stable from the first 5 minutes to the last 5 minutes, and the phase shifts in all frequency bands and *Mx* were not significantly different between the 5- and 10-minute recordings. Moreover, the AUCs of ROC curves for identifying patients with stroke were not significantly different in phase shift in all frequency bands and *Mx* between the 5- and 10-minute recordings. Therefore, in the study of stroke, the application of dCA based on spontaneous CBFV and BP changes would not be significantly different between the 5- and 10-minute recordings. A study of 16-minute recording revealed that ARI, *Mx*, and phase shift would be stable after 3, 6, and 5 minutes, respectively [19]. A 5-minute recording length may be sufficient for dCA assessment. However, the gain and coherent were slightly but significantly higher in 5-minute than those in 10-minute recording; the reasons are unclear and need further investigations.

The higher coherence and gain of the 5-minute recording than those of the 10-minute recording might be explained by methodological issues. In this study, we used raw waveform of CBFV and BP for dCA analysis, and the “physiocal” of the Finapres device remained active throughout the recording period. Deegan et al. reported that gain and coherence but not phase shift would decrease as signal artifacts increase when using raw waveform of CBFV and BP for TFA [27], which is similar to our findings. In this study, average of 4 to 6 “physiocal” occurred in a 5-minute recording, and the number of “physiocal” doubled in a 10-minute recording; however, the ratio of artifacts to signals in time is the same between 5- and 10-minute recordings; hence, other mechanisms that decrease gain and coherence may also exist. In Deegan's study of TFA estimated from 1 to 5 minutes of recordings, there was a trend that gain and coherence but not phase shift decreased as the data length increased, and the gain and coherence but not phase shift in a 5-minute recording were significantly smaller than those in a 1-minute recording [27]. In this study, the gain and coherence were not different between the first and last 5 minutes but were smaller in the 10 minutes than in each of the first and last 5 minutes. Therefore, it is possible that gain and coherence decrease as the data length increases which is a nature of TFA rather than a physiological phenomenon. The stability of gain and coherence in TFA has not been tested in large scale or discussed, and it seems that in a 10-minute time scale, gain and coherence may not be stable according to Deegan's and our findings. In previous studies of dCA in stroke, gain has not been reported to differ between patients and controls [12, 14, 21, 28]. If gain is an unstable dCA index, it would be difficult to correlate gain with other physiologic or clinical variables. In the ROC curve analysis in this study, gain was not valid for identifying patients with stroke. However, it is possible that gain would stabilize in a larger time scale for more than 10 minutes, and further investigations are warranted.

This study has limitations. First, we compared the validity only in identifying stroke between 5- and 10- minute recordings, and whether the studies of other diseases could benefit from a longer recording length is unclear. Second, it is unclear whether a recording period longer than 10 minutes would yield a result different from ours. Third, we used raw waveform of CBFV and BP for dCA analysis, and the results of using beat-to-beat data need further investigations.

## 5. Conclusion

The *Mx* and phase shift assessed under spontaneous CBFV and BP changes are not significantly different between 5- and 10-minute recordings and have the same validity in the study of stroke. However, gain and coherence are higher in the 5-minute recording compared to those in the 10-minute recording. A unified recording length in a single study or between studies could minimize the influences of time-dependent variables.

## Figures and Tables

**Table 1:** Clinical characteristics of the subjects.

	Stroke (+)	Stroke (−)	*P* value
	*n* = 37	*n* = 51	
Age (range)	56 ± 11 (33−80)	47 ± 14 (20−67)	0.001
Sex: male	28 (76%)	18 (35%)	<0.001
Comorbidities			
Hypertension	26 (70%)	13 (25%)	<0.001
Diabetes	14 (38%)	7 (14%)	0.012
Hyperlipidemia	23 (62%)	23 (45%)	0.134
NIHSS (range)	3 ± 3 (0−15)		
Stroke etiology			
LAA	15 (40.5%)		
SVD	22 (59.5%)		

LAA: large artery atherosclerosis; NIHSS: National Institute of Health Stroke Scale, obtained on the day of dCA assessment; and SVD: small vessel disease.

**Table 2:** Agreements between dCA assessed for 5 and 10 mins.

All subjects (*n* = 88)	The first 5 mins, median (IQR)	The last 5 mins, median (IQR)	10 mins, median (IQR)	Mean difference ± 95% of agreement between the first 5 mins and 10 mins	Intraclass correlation coefficient between the first 5 mins and 10 mins (95% CI)
*Mx*	0.37 (0.18–0.48)	0.34 (0.11–0.52)	0.35 (0.15–0.48)	0.02 ± 0.19	0.93 (0.90–0.96)
Phase Shift (Degree)					
VLF (0.02–0.07 Hz)	55 (41–74)	56 (41–73)	57 (45–73)	−0.3 ± 28.1	0.87 (0.81–0.91)
LF^†^ (0.07–0.20 Hz)	39 (29–49)	36 (23–50)	36 (26–47)	0.6 ± 42.0	0.74 (0.62–0.82)
HF^†^ (0.20–0.50 Hz)	9 (−8–19)	9 (−3–22)	6 (−5–17)	−0.1 ± 37.6	0.76 (0.65–0.84)
Gain (cm/s/mmHg)					
VLF (0.02–0.07 Hz)	0.43 (0.32–0.66)	0.47 (0.33–0.69)	0.30 (0.30–0.61)^*∗*§^	0.05 ± 0.29^#^	0.94 (0.90–0.96)
LF^†^ (0.07–0.20 Hz)	0.48 (0.34–0.65)	0.47 (0.35–0.62)	0.38 (0.28–0.55)^*∗*§^	0.11 ± 0.27^#^	0.77 (0.37–0.89)
HF^†^ (0.20–0.50 Hz)	0.49 (0.37–0.66)	0.48 (0.38–0.65)	0.38 (0.28–0.53)^*∗*§^	0.14 ± 0.28^#^	0.78 (0.25–0.91)
Coherence					
VLF (0.02–0.07 Hz)	0.46 (0.29–0.62)	0.46 (0.24–0.63)	0.41 (0.25–0.59)^*∗*§^	0.04 ± 0.22^#^	0.80 (0.70–0.87)
LF (0.07–0.20 Hz)	0.33 (0.21–0.46)	0.30 (0.19–0.44)	0.25 (0.15–0.40)^*∗*§^	0.06 ± 0.14^#^	0.86 (0.44–0.95)
HF (0.20–0.50 Hz)	0.23 (0.16–0.29)	0.21 (0.16–0.27)	0.15 (0.10–0.21)^*∗*§^	0.07 ± 0.13^#^	0.69 (0.11–0.87)

^*∗*^ *P* < 0.05 compared to the first 5 mins; ^§^*P* < 0.05 compared to the last 5 mins; ^#^*P* < 0.05 mean difference = 0; ^†^*n* = 78; CI: confidence interval.

**Table 3:** The accuracy of identifying stroke patients in all subjects in the first 5 mins and 10 mins of recordings.

All subjects (*n* = 88)	Area under the ROC curve (95% confidence interval)	
	5 mins	10 mins
*Mx*	0.714 (0.607–0.805)^*∗*^	0.719 (0.613–0.810)^*∗*^
Phase shift (degree)		
VLF (0.02–0.07 Hz)	0.707 (0.600–0.799)^*∗*^	0.716 (0.610–0.807)^*∗*^
LF^†^ (0.07–0.20 Hz)	0.557 (0.439–0.670)	0.568 (0.450–0.681)
HF^†^ (0.20–0.50 Hz)	0.507 (0.391–0.622)	0.510 (0.395–0.625)
Gain (cm/s/mmHg)		
VLF (0.02–0.07 Hz)	0.531 (0.422–0.638)	0.548 (0.438–0.654)
LF^†^ (0.07–0.20 Hz)	0.560 (0.443–0.673)	0.511 (0.395–0.626)
HF^†^ (0.20–0.50 Hz)	0.599 (0.481–0.708)	0.503 (0.388–0.619)

^*∗*^ *P* < 0.05 AUC = 0.5; all areas under the ROC curves were not significantly different between 5 mins and 10 mins; ^†^*n* = 78.

## Supplementary Materials

Supplementary Materials Supplementary Table Agreements between dCA assessed for 5 and 10 minutes in (A) patients with stroke and (B) controls.
