# Supplementary material for: Comparing Different Recording Lengths of Dynamic Cerebral Autoregulation: 5 versus 10 Minutes
Source: Biomed Res Int. 2018 Jan 31;2018:7803426. doi: 10.1155/2018/7803426 (PMC5831790; doi:10.1155/2018/7803426)
Supplement: Supplementary Materials — Supplementary Table Agreements between dCA assessed for 5 and 10 minutes in (A) patients with stroke and (B) controls. [file 7803426.f1.docx]

| **Supplementary Table.  Agreements between dCA assessed for 5 and 10 minutes in (A)Patients with stroke (B) controls** | | | | | |
| --- | --- | --- | --- | --- | --- |
| **(A)** |  |  |  |  |  |
| **Patients with stroke (n= 37)** | | The first 5 mins, median (IQR) | The last 5 mins, median (IQR) | 10 mins, median (IQR) | Mean Difference ± 95% of Agreement between the first 5 mins and 10 mins |
| **Mx** | | 0.43 (0.33 -0.60) | 0.46 (0.27 - 0.57) | 0.40 (0.32 - 0.56) | 0.02 ± 0.17 |
| **Phase Shift (Degree)** | **VLF (0.02-0.07 Hz)** | 49 (34 - 62) | 52 (39 - 62) | 50 (38 - 63) | -1.1 ± 31.7 |
|  | **LF† (0.07-0.20 Hz)** | 42 (19 - 60) | 42 (30 - 60) | 36 (27 - 61) | -1.0 ± 42.3 |
|  | **HF† (0.20-0.50 Hz)** | 12 (-12 - 19) | 13 (-1 -24) | 6 (-6 - 18) | -3.4 ± 43.8 |
| **Gain (cm/s/mmHg)** | **VLF (0.02-0.07 Hz)** | 0.41(0.32 - 0.70) | 0.46 (0.31 - 0.70) | 0.45 (0.30 - 0.60)*§ | 0.04 ± 0.34 |
|  | **LF† (0.07-0.20 Hz)** | 0.43 (0.34 - 0.63) | 0.47 (0.40 - 0.58) | 0.38 (0.31 - 0.45)*§ | 0.09 ± 0.28# |
|  | **HF† (0.20-0.50 Hz)** | 0.43 (0.34 - 0.60) | 0.49 (0.37 - 0.67) | 0.37 (0.27 - 0.55)*§ | 0.11 ± 0.35# |
| **Coherence** | **VLF (0.02-0.07 Hz)** | 0.47 (0.28 - 0.66) | 0.48 (0.35 - 0.61) | 0.43 (0.28 - 0.59) | 0.03 ± 0.24 |
|  | **LF (0.07-0.20 Hz)** | 0.28 (0.19 - 0.43) | 0.30 (0.21 - 0.40) | 0.21 (0.13 - 0.34)*§ | 0.05 ± 0.16# |
|  | **HF (0.20-0.50 Hz)** | 0.23 (0.14 - 0.33)§ | 0.20 (0.15 -0.25)* | 0.14 (0.09 - 0.23)*§ | 0.08 ± 0.16# |
| *P < 0.05 compared to the first 5 mins; §P<0.05 compared to the last 5 mins; #P < 0.05 mean difference = 0; †n=32; CI: confidence interval; SD: standard deviation | | | | | |

| **(B)** |  |  |  |  |  |
| --- | --- | --- | --- | --- | --- |
| **Controls (n=51)** | | The first 5 mins, median (IQR) | The last 5 mins, median (IQR) | 10 mins, median (IQR) | Mean Difference ± 95% of Agreement between the first 5 mins and 10 mins |
| **Mx** | | 0.32 (0.07 - 0.43) | 0.28 (0.02 - 0.46) | 0.30 (0.02 - 0.41) | 0.01 ± 0.20 |
| **Phase Shift (Degree)** | **VLF (0.02-0.07 Hz)** | 59 (51 - 82) | 59 (42 - 76) | 65 (48 - 82) | 0.3 ± 25.5 |
|  | **LF† (0.07-0.20 Hz)** | 38 (29 - 46) | 32 (22 - 43) | 36 (26 - 44) | 1.6 ± 27.2 |
|  | **HF† (0.20-0.50 Hz)** | 8 (-2 - 16) | 7 (-6 - 18) | 8 (-5 - 16) | 1.9 ± 32.9 |
| **Gain (cm/s/mmHg)** | **VLF (0.02-0.07 Hz)** | 0.44 (0.31 - 0.64) | 0.49 (0.36 - 0.68) | 0.39 (0.29 - 0.60)*§ | 0.06 ± 0.25# |
|  | **LF† (0.07-0.20 Hz)** | 0.55 (0.33 - 0.68) | 0.49 (0.32 - 0.78) | 0.42 (0.26 -0.57)*§ | 0.12 ± 0.25# |
|  | **HF† (0.20-0.50 Hz)** | 0.55 (0.38 - 0.69) | 0.47 (0.38 - 0.65) | 0.38 (0.29 - 0.53)*§ | 0.16 ± 0.23# |
| **Coherence** | **VLF (0.02-0.07 Hz)** | 0.43 (0.31 - 0.61) | 0.42 (0.22 - 0.66) | 0.38 (0.24 - 0.60)* | 0.05 ± 0.21# |
|  | **LF (0.07-0.20 Hz)** | 0.38 (0.25 - 0.47)§ | 0.30 (0.18 - 0.51)* | 0.27 (0.15 - 0.41)*§ | 0.07 ± 0.12# |
|  | **HF (0.20-0.50 Hz)** | 0.22 (0.16 - 0.28) | 0.24 (0.17 - 0.28) | 0.17 (0.12 - 0.21)*§ | 0.06 ± 0.10# |
| *P < 0.05 compared to the first 5 mins; §P<0.05 compared to the last 5 mins; #P < 0.05 mean difference = 0; †n=46; CI: confidence interval; SD: standard deviation | | | | | |
